# Peripapillary retinal nerve fiber layer thinning in patients with progressive supranuclear palsy

**DOI:** 10.1007/s00415-021-10936-5

**Published:** 2021-12-18

**Authors:** Kyung Ah Woo, Joo Young Shin, Heejung Kim, Jeeyun Ahn, Beomseok Jeon, Jee-Young Lee

**Affiliations:** 1 Department of Neurology, Seoul Metropolitan Government-Seoul National University Boramae Medical Center, Seoul, Republic of Korea; 2 Department of Ophthalmology, Seoul Metropolitan Government-Seoul National University Boramae Medical Center, Seoul, Republic of Korea; 3 Institute of Radiation Medicine, Medical Research Center, Seoul National University, Seoul, Republic of Korea; 4 Department of Neurology, Seoul National University Hospital, Seoul, Republic of Korea; 5 Seoul National University College of Medicine, Seoul, South Korea; 6 Department of Nuclear Medicine, Seoul Metropolitan Government-Seoul National University Boramae Medical Center, Seoul, Republic of Korea

**Keywords:** Progressive supranuclear palsy (PSP), Optical coherence tomography (OCT), Retinal Nerve Fiber Layer (RNFL), Magnetic Resonance Imaging (MRI)

## Abstract

**Objectives:**

To investigate peripapillary retinal nerve fiber layer (pRNFL) changes in patients with progressive supranuclear palsy (PSP).

**Methods:**

We included 21 PSP patients (36 eyes) who underwent peripapillary optical coherence tomography (OCT) scans at 2.5 ± 1.3 years of disease, without ophthalmologic co-morbidities. We compared pRNFL thicknesses in PSP eyes with age-matched 22 controls (22 eyes) using generalized estimating equation model adjusting for intra-subject inter-eye correlations, age and sex. We also analyzed the correlation between the pRNFL thickness and clinical severity using Spearman’s correlation. In twelve PSP patients with 3 T brain MRI volumetric scan within 1 year of OCT exam, we investigated the correlation between the pRNFL thickness and brain atrophy using Pearson’s correlation.

**Results:**

PSP patients had global pRNFL thinning compared to controls (beta = − 6.436, *p* = 0.025). Global pRNFL thickness correlated with Hoehn & Yahr stages (*r* = − 0.487, *p* = 0.025), and nasal pRNFL thinning showed a trend of correlation (uncorrected *p* < 0.05). Exploratory correlation analysis between global pRNFL thickness and nonmotor items in the PSP rating scale showed a trend toward association with sleep disturbances (uncorrected *p* = 0.008) and urinary incontinence (uncorrected *p* = 0.031), although not significant after Bonferroni correction (all 28 items). The patients had significant atrophy in the posterior cingulate cortex, third ventricle, pallidum, and midbrain with reduced midbrain-to-pons ratio, but no correlation was found between pRNFL thickness and brain volumes.

**Conclusion:**

The pRNFL seems to be affected in PSP, which is more severe with advanced disease stages. Retinal investigation in a larger longitudinal cohort would help elucidate the pathophysiological role of retinal thinning in PSP.

**Supplementary Information:**

The online version contains supplementary material available at 10.1007/s00415-021-10936-5.

## Introduction

Progressive supranuclear palsy (PSP) is a form of atypical parkinsonism characterized by 4-repeat tau neuropathology. Patients with the classic PSP-Richardson phenotype exhibit vertical supranuclear gaze palsy and prominent axial symptoms, but the clinical presentations in the early stages of PSP can be highly variable [1]. Still, there is an unmet need for a clinical biomarker that enables early recognition of PSP and reflects the disease progression.

Visual disturbances frequently appear from the early or prediagnostic stages of PSP [2, 3]. Common visual manifestations include slowing of vertical saccades and blurred vision, diplopia, photophobia, and burning eyes. Indeed, visually triggered orientation and pupil responses are served by retinal input to the superior colliculus and pretectal olivary nucleus in the midbrain, where the PSP pathology predominates [4, 5]. Neurophysiologic studies in PSP revealed changes in event-related visual evoked potentials and visuospatial deficits, suggesting the possible alteration of the visual afferent pathway [6, 7].

The retina is the only extension of the central nervous system that can be directly imaged in vivo. The peripapillary retinal nerve fiber layer (pRNFL) consists of ganglion cell axons that directly connect to the brain, and its thickness can be noninvasively measured by optical coherence tomography (OCT). The pRNFL represents the most proximal part of the visual afferent pathway; therefore, measuring its thickness change is reproducible to detect retinal axonal degeneration. Studies have shown pRNFL thinning in patients with neurodegenerative diseases, such as Parkinson’s disease (PD), multiple system atrophy (MSA), and Alzheimer’s disease (AD) [8–10]. In a few studies with PSP, the pRNFL is shown to be thinner in PSP than healthy controls or PD [11–13], and one study suggested no correlation with disease severity or duration [13]. However, these studies included only small samples or did not engage a standard analytic model following the APOSTEL recommendations [14]. Furthermore, brain volumetric measures such as midbrain, pons, frontal lobe, and third ventricle have been shown to correlate with clinical disease progression of PSP [15–17], but no study has examined the relationship between these representative regions of brain atrophy and retinal changes in PSP.

In this context, the purpose of this study was to evaluate if pRNFL thinning was present in clinically diagnosed PSP patients, and to explore the association between pRNFL thickness and the clinical symptoms of PSP. We also investigated the relationship between pRNFL thinning and brain atrophy in regions specific to PSP.

## Materials and methods

### Study population

Consecutive patients who visited the movement disorders clinic at Seoul National University Boramae Medical Center (SNUBMC) between 2012 and 2018, and who were clinically diagnosed with PSP by a movement disorders specialist (J.Y.L.) and underwent peripapillary OCT scan and ophthalmologic examinations by an ophthalmologic specialist (J.A.) were eligible for this study. Thus, bedridden patients who were unable to undergo OCT were not included in this study. The eyes with comorbid ophthalmologic pathologies that could affect the pRNFL findings on OCT were excluded from the analysis, such as glaucomatous optic neuropathy, optic neuropathies of other causes, age-related macular degeneration, diabetic retinopathy, epiretinal membrane, and retinal artery or vein occlusion.

For participants with PSP, clinical and demographic information including age, sex, duration of PSP at the time of the OCT scan, and clinical severity ratings assessed by the Hoehn and Yahr (HY) stages [18], the Progressive Supranuclear Palsy Rating Scale (PSPRS) [19], and the Mini-Mental State Examination (MMSE) [20] scores at the time of the OCT scan were collected. The diagnosis of PSP in these study subjects was reviewed according to the 2017 Movement Disorder Society Criteria [21]. Healthy control data of peripapillary OCT scan in our center and healthy control MRI database of our center was used for comparison analysis as described below. The healthy controls were prospectively recruited during the same study period among those who visited our center for regular health checkups and had no neurological disease.

The Institutional Review Board of the SNUBMC approved this study and informed consent was waived by the IRB due to the retrospective analysis of the study protocol.

### Optical coherence tomography

OCT scans were performed at the ophthalmology department by an experienced ophthalmologic technician, with careful inspection during image acquisition for any correctable artifacts such as motion artifacts and rescanning if any occurred. Any images with poor quality or severe artifacts interfering with the analysis were excluded from the study. Participants underwent Spectralis OCT (Heidelberg Engineering, Heidelberg, Germany) and Spectral OCT/SLO (Ophthalmic Technologies Inc, Toronto, Canada) as previously described [22]. For the pRNFL measure, a circular scan centered on the optic nerve head with a diameter of approximately 3.46 mm for Spectralis OCT and 3.4 mm for Spectral OCT/SLO was obtained and automatically segmented into superior, temporal, nasal, and inferior areas by the machine’s software. We collected automatically measured pRNFL thicknesses in each of the segmented quadrants and the global peripapillary area.

### Estimation of ROI volumes in brain MRI

For subjects who underwent 3T brain MRI with a 32-channel phased-array head coil (3T Discovery 750w, GE Medical Systems Milwaukee, WI, USA) within 1 year of OCT scan, we collected 3D T1 volumetric data. The acquisition parameters were as follows: echo time (TE), 3.0 ms; repetition time (TR), 8.0 ms; voxel size, 1 × 1 × 1 mm^3^; acquisition matrix, 256 × 256; field of view (FOV), 25.6 mm; number of excitations (NEX), 1; flip angle, 12°, and slice thickness, 1 mm with no gap. The data underwent bias field correction and whole-brain parcellation with the geodesic information flow (GIF) algorithm.

Preprocessing was performed using Statistical Parametric Mapping (SPM12, Wellcome Department of Imaging Neuroscience, London, UK, http://www.fil.ion.ucl.ac.uk/spm) implemented in Matlab 9.5 (The MathWorks, Inc., Natick, MA, USA). ROI extraction was performed using the Computational Anatomy Toolbox (CAT 12) (http://www.neuro.unijena.de/cat) embedded in SPM12. Each anatomical image was segmented into grey matter (GM), white matter (WM) and cerebrospinal fluid (CSF) and non-linearly normalized to a standard stereotactic space using the DARTEL algorithm. The preprocessed and normalized images were parcellated based on the Automated Anatomical Labelling (AAL3) template [23, 24]. The volumes of 15 brain regions of interest (ROI) were determined, including the grey matter, white matter, thalamus, anterior and posterior cingulate cortices, putamen, pallidum, amygdala, cerebellum, third ventricle, midbrain, pons, superior cerebellar peduncle, and medulla. The third ventricle and midbrain volumes were particularly included, because these were reported to be reliable markers of disease progression in PSP [15–17]. We segmented the volumes of the superior cerebellar peduncle, midbrain, pons, and medulla using a customized module in FreeSurfer (https://surfer.nmr.mgh.harvard.edu/fswiki/BrainstemSubstructures), and calculated the midbrain/pons ratio. Total intracranial volume (TIV) was adjusted for when analyzing the ROI volumes.

### Statistical analysis

Data are presented as mean (standard deviation; SD) unless specified otherwise. The normality of the data was assessed with the Shapiro–Wilk test. We compared the demographic data of PSP patients and healthy controls using *t* test or Mann–Whitney for continuous variables and *χ*^2^ test or Fisher’s exact for categorical variables. The pRNFL measures of the PSP subtypes were compared by the Kruskal–Wallis test.

To compare eye-specific pRNFL measures between PSP patients and healthy controls, we used a generalized estimating equation (GEE) model with an exchangeable structure based on a linear model, controlling for within-patient inter-eye correlations and the effect of age and sex on pRNFL thickness. In patients with PSP, Spearman’s correlation analysis was performed to explore the relationship between the pRNFL thickness and clinical severity assessed by HY stages and the PSPRS, along with MMSE. When both eyes from one patient were included, we opted for the averaged value of both eyes for correlation analysis. To explore the relationship of pRNFL thinning with nonmotor symptoms of PSP, we also performed an exploratory correlation analysis between the pRNFL thickness and the nonmotor-related items in the PSPRS part 1 using Spearman’s method.

Comparison of brain atrophy in predefined ROIs between the PSP-MRI group and the healthy control database was made using the analysis of covariance (ANCOVA) with age adjustment. We performed correlation analyses between the brain ROIs with a significant reduction in the PSP-MRI group compared to the healthy controls and the averaged pRNFL measures by the Pearson’s partial correlation method controlling for age.

All statistical analyses were performed using SPSS 23.0 (SPSS Inc, Chicago IL) and R software, version 3.6.0. (R project for Statistical Computing) with a limit of significance set at 0.05.

## Results

### Characteristics of the participants

25 PSP patients were eligible for this study. Among the 50 eyes examined, 14 eyes of 10 patients were excluded because of ophthalmologic comorbidities following the exclusion criteria described above. A total of 21 PSP patients (36 eyes) and 22 healthy controls (22 eyes) were included in the final analysis.

The baseline demographic characteristics of the subjects included in this study are presented in Table 1. There were no significant differences in age and sex between the PSP group and the controls (*p* = 0.162, 0.864, respectively).

**Table 1:** Demographic characteristics of the PSP patients and controls

	PSP (*n* = 21, 36 eyes)	PSP-MRI (*n* = 12, 22 eyes)	Control (*n* = 22, 22 eyes)	*p* value*
Age, years	71.8 (5.2)	70.1 (4.9)	69.5 (5.4)	0.162
Male, *n* (%)	9 (42.9)	2 (16.7)	10 (45.5)	0.864
Disease duration, years	2.5 (1.3)	2.2 (1.3)		
Hoehn and Yahr stage, *n* (%)				
2 or 2.5	8 (38.1)	4 (33.3)		
3	5 (23.8)	3 (25.0)		
4	8 (38.1)	5 (41.7)		
MMSE	21.8 (5.4)	21.3 (6.3)		
PSP Rating Scale	28.3 (11.9)	27.4 (14.5)		
Diagnostic certainty, *n* (%)				
Probable PSP	17 (81.0)	8 (66.7)		
Possible PSP	4 (19.0)	4 (33.3)		
Diagnostic classification, *n* (%)				
PSP-RS	9 (42.9)	3 (25.0)		
PSP-PGF	9 (42.9)	7 (58.3)		
PSP-P	3 (14.2)	2 (16.7)		

Data presented as mean (SD) unless otherwise specified; n represents the number of subjects

*Comparison between the PSP group and the controls

In the 21 patients with PSP, the mean disease duration was 2.5 years, and the HY stages ranged from 2 to 4. The mean MMSE score was 21.8 (SD 5.4) and the mean PSPRS score was 28.3 (SD 11.9).

The diagnostic certainty and subclassifications of PSP were provided in Table 1. Among the 21 PSP patients, nine PSP-RS (PSP with Richardson’s syndrome), 9 PSP-PGF (PSP with progressive gait freezing), and 3 PSP-P (PSP with predominant parkinsonism) subtypes were identified according to the MDS diagnostic criteria for PSP [21]. Regarding the diagnostic certainty, four had possible PSP, all with PSP-PGF, and 17 had probable PSP. All of the probable PSP patients had O1 or O2 ocular motor dysfunction in the MDS diagnostic criteria. In the four patients diagnosed as possible PSP-PGF, brain MRI showed no vascular lesion or signal changes, excluding the possibility of vascular gait disorder.

### Peripapillary RNFL thickness in PSP and comparison to controls

**Fig. 1:**
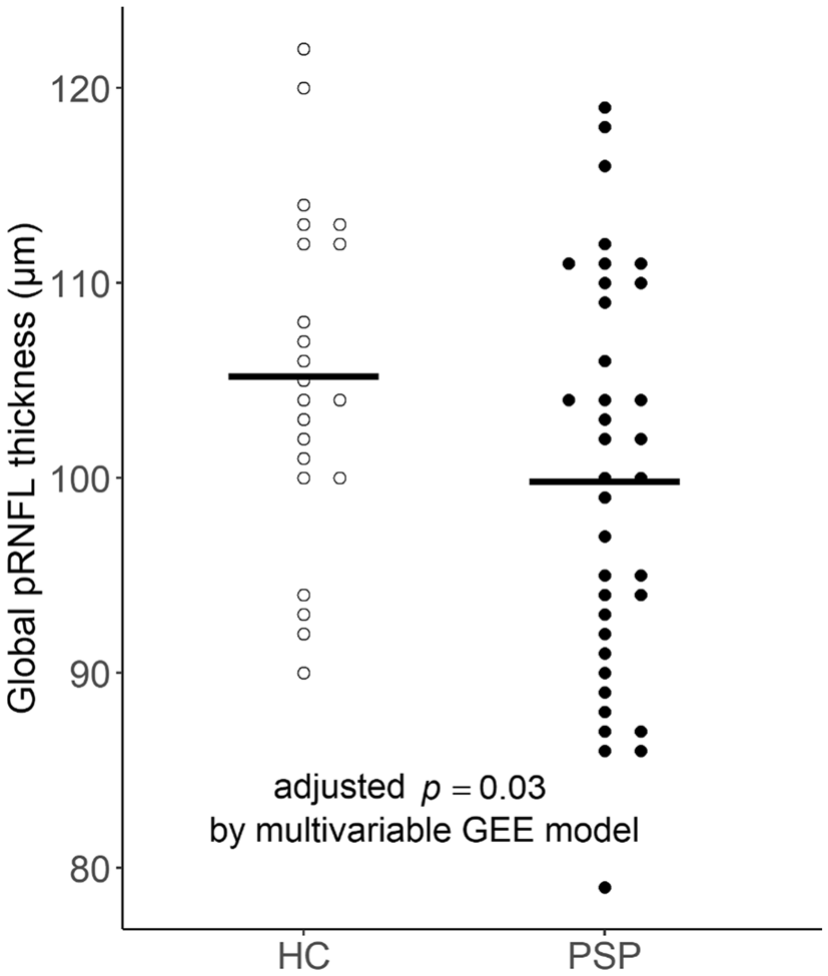
Peripapillary RNFL thickness in patients with PSP (36 eyes) and healthy controls (HC, 22 eyes). The bars represent the mean value of eye-specific peripapillary RNFL thickness. pRNFL, peripapillary retinal nerve fiber layer

The peripapillary RNFL thickness of PSP patients and healthy controls are shown in Fig. 1. The mean value of the eye-specific global pRNFL thickness was 99.83 μm in 36 eyes of PSP patients (range 79–119; SD 10.27), and 105.23 μm in 22 eyes of healthy controls (range 90–122; SD 8.66). For each of the PSP subtypes, the mean global pRNFL thickness in the PSP-RS subtype was 103.27 μm (15 eyes; range 79–116; SD 11.30), in PSP-PGF type 98.69 μm (16 eyes; range 88–119; SD 9.08), and in PSP-P type 93.20 μm (5 eyes; range 86–104; SD 7.92). No statistically significant difference was found among the global pRNFL thickness of the three PSP subtypes (*p* = 0.091).

In the univariable GEE analysis, PSP patients had significant global pRNFL thinning compared to the healthy controls (beta = − 6.436, *p* = 0.025; Table 2). The difference was still significant in the multivariable GEE analysis additionally considering age and sex (beta = − 5.718, *p* = 0.030). In the post-hoc analysis for each retinal peripapillary quadrant (superior, nasal, temporal, and inferior), the pRNFL thinning tend to be prominent in the inferior sector (by multivariable GEE analysis adjusted for age and sex, beta = − 10.486, *p* = 0.039), although the significance was lost after Bonferroni-correction (corrected *p* = 0.156).

**Table 2:** Comparison of retinal nerve fiber layer thickness between PSP patients (*n* = 21, 36 eyes) and controls (*n* = 22, 22 eyes)

Variable	Beta (SE; 95% CI)	*p* value
2-1. Estimated mean differences in global RNFL thickness on univariable GEE		
Age, 1-year increase	− 0.378 (0.2888; − 0.944, 0.188)	0.190
Sex, male vs. female	2.273 (2.9305; − 3.471, 8.017)	0.438
PSP vs. control	− 6.436 (2.8256; − 11.884, − 0.808)	0.025
2-2. Estimated mean differences in global RNFL thickness on multivariable GEE		
Intercept	121.782 (18.9733; 84.595, 158.969)	< 0.001
Age, 1-year increase	− 0.251 (0.2660; − 0.772, 0.271)	0.346
Sex, male vs. female	1.908 (2.7617; − 3.504, 7.321)	0.490
PSP vs. control	− 5.718 (2.6333; − 10.879, − 0.556)	0.030

Retinal quadrant	Beta (SE; 95% CI)	*p* value*	Corrected *p* value^†^
2-3. Post hoc analysis for RNFL thickness of retinal quadrants in PSP patients vs. controls			
Superior	− 4.250 (3.9891; − 12.068, 3.569)	0.287	
Temporal	− 5.472 (3.2589; − 11.859, 0.916)	0.093	
Nasal	− 1.327 (3.5296; − 8.245, 5.590)	0.707	
Inferior	− 10.486 (5.0738; − 20.431, − 0.542)	0.039	0.156

*Adjusted for age and sex with multivariable generalized estimating equations

^†^Bonferroni-corrected *p* values for multiple comparisons of the four retinal quadrants are shown

### Correlation between peripapillary RNFL thickness and clinical severity in PSP

**Fig. 2:**
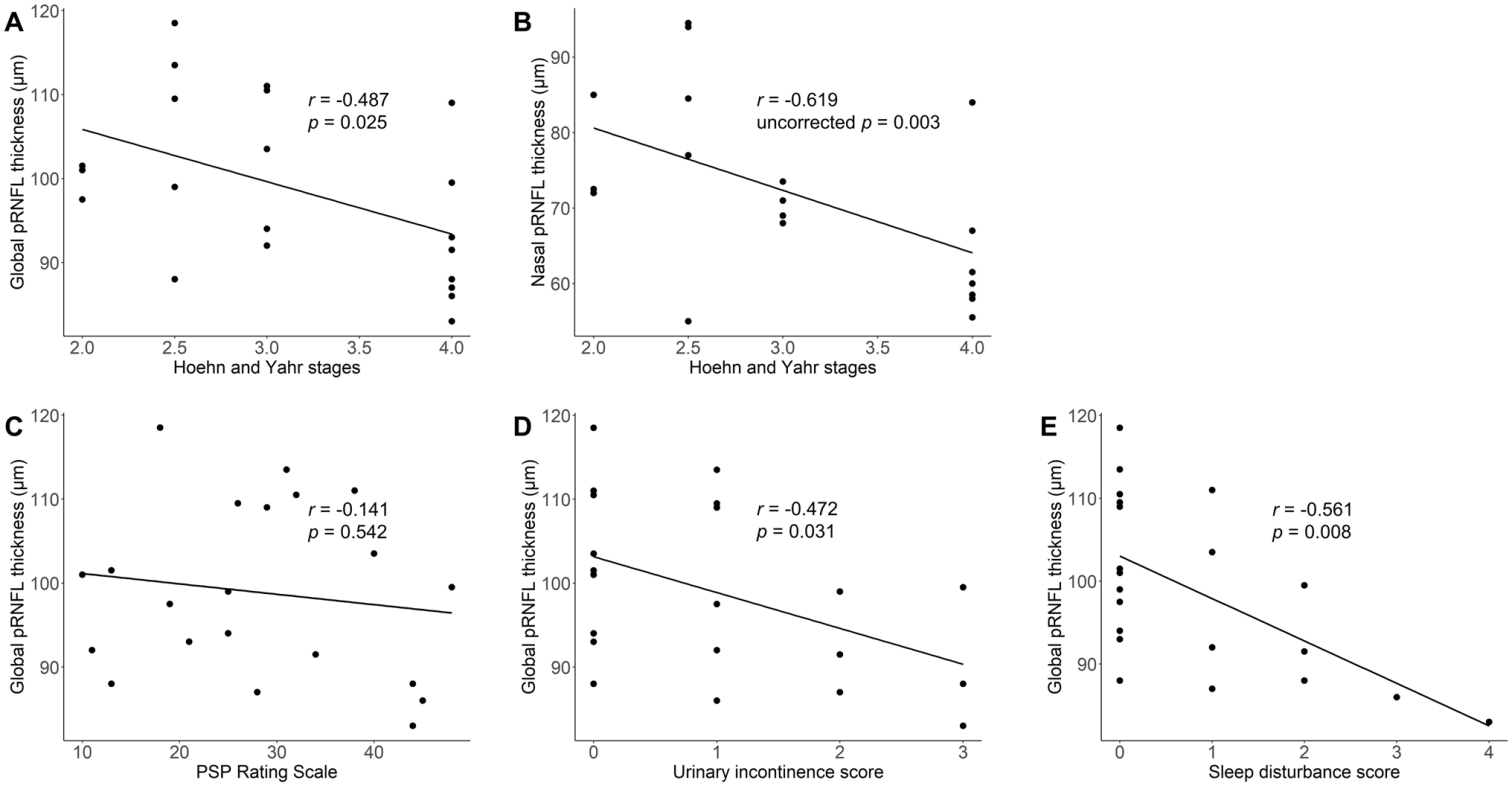
Peripapillary RNFL thickness and clinical severity in patients with PSP. The scatterplot shows the relationship between the peripapillary retinal nerve fiber layer (pRNFL) thickness, Hoehn and Yahr (HY) stages, and the PSP Rating Scale (PSPRS) in the patients with PSP. The *r * and *p *values are based on Spearman’s correlation analysis. When both eyes of 1 patient were included, the mean value of pRNFL thickness was used. HY stages were negatively correlated with global and nasal pRNFL thicknesses (**A**, **B**). No significant correlation was found between the pRNFL thickness and the PSPRS total score (**C**). An exploratory correlation analysis with nonmotor symptoms assessed by the related items of the PSPRS showed a tendency of negative correlation between the global pRNFL thickness and the urinary incontinence score, and the sleep disturbance score (**D**, **E**)

Global pRNFL thinning tended to be more severe in advanced HY stages (*r* = − 0.487, *p* = 0.025 by Spearman’s correlation analysis; Fig. 2A). The correlation was also significant in the subgroup of 9 PSP-RS patients (*r* = − 0.704, *p* = 0.034), and a tendency of negative correlation was observed in the 9 PSP-PGF patients (*r* = − 0.338, *p* = 0.374). For the post-hoc analysis of peripapillary quadrants, there was a negative correlation between the nasal pRNFL thickness and HY stages (*r* = − 0.619, *p* = 0.003; Bonferroni-corrected *p* = 0.012; Fig. 2B). No significant correlation was found between global pRNFL thickness and the MMSE scores (*r* = − 0.252, *p* = 0.271), disease duration (*r* = − 0.108, *p* = 0.640), or the PSPRS (*r* = − 0.141, *p* = 0.542; Fig. 2C).

In the exploratory correlation analysis between global pRNFL thickness and nonmotor-related items in the PSPRS, a tendency of possible negative correlation was found with urinary incontinence (*r* = − 0.472, uncorrected *p* = 0.031; Fig. 2D) and sleep disturbance (*r* = − 0.561, uncorrected *p* = 0.008; Fig. 2E). No significant correlation was found between the pRNFL thickness and the PSPRS ocular motor sum score of items 14 to 16. The full results of exploratory correlation analysis between global pRNFL thickness and individual items of PSPRS are presented in the Supplementary Table 1. When Bonferroni correction was applied for multiple comparisons, no items reached the significance level.

### Regions of brain atrophy in PSP and correlation with pRNFL thickness

Volumetric MRI were analyzed in 12 PSP patients (PSP-MRI group represented in Table 1). The mean time interval between brain MRI and peripapillary OCT scans was 81.17 days (SD 70.06). Comparison of the clinical characteristics of the total PSP subjects and the PSP-MRI subgroups showed no significant difference in age (*p* = 0.359), sex (*p* = 0.249), disease duration (*p* = 0.539), diagnostic certainty (*p* = 0.420), HY stages (*p* = 0.795), MMSE (*p* = 0.838) and PSPRS (*p* = 0.853). The healthy control MRI database of our center consisted of 28 control subjects with mean age of 70.03 (SD 4.58), and there were no differences in age (*p* = 0.971) and sex (*p* = 0.451) between the MRI controls and the PSP-MRI group.

Compared to healthy controls, PSP patients had third ventricle enlargement (*p* = 0.033) along with the atrophy of grey matter (*p* = 0.014), white matter (*p* = 0.035), posterior cingulate cortex (*p* = 0.045), pallidum (*p* = 0.044), midbrain (*p* = 0.006), and decreased midbrain/pons ratio (*p* = 0.035), as shown in Table 3. Correlation analysis between these brain regional atrophy and global pRNFL thickness revealed no significant relationship (*p* > 0.05 for all with or without age adjustment). An additional exploratory analysis between brain atrophy and the PSPRS ocular motor sum scores showed a tendency of negative correlation between the ocular motor dysfunction severity and midbrain volume normalized to the total intracranial volume (*r* = − 0.614, uncorrected *p* = 0.034).

**Table 3:** Brain ROI volume and correlation with global averaged peripapillary RNFL thickness in PSP patients

Brain ROI volume (ROI/TIV, %)	PSP-MRI (*N* = 12)	HC (*N* = 28)	*p* value	Correlation between brain ROI volume and global pRNFL thickness			
				Pearson correlation coefficient	*p* value	Partial correlation coefficient with age adjustment	*p* value
Medulla	0.286 (0.032)	0.295 (0.024)	0.379				
Pons	0.875 (0.145)	0.919 (0.081)	0.237				
Superior cerebellar peduncle	0.015 (0.004)	0.015 (0.002)	0.967				
Midbrain	0.346 (0.049)	0.382 (0.027)	0.006	− 0.043	0.894	− 0.031	0.928
Midbrain/Pons ratio	0.398 (0.032)	0.416 (0.019)	0.035	− 0.235	0.463	− 0.237	0.483
Grey matter	37.941 (1.835)	39.657 (1.927)	0.014	− 0.271	0.395	− 0.272	0.418
White matter	30.008 (3.139)	31.560 (1.468)	0.035	0.340	0.280	0.397	0.227
Thalamus	0.650 (0.052)	0.669 (0.071)	0.422				
Anterior cingulate cortex	0.511 (0.054)	0.517 (0.048)	0.731				
Posterior cingulate cortex	0.495 (0.060)	0.533 (0.048)	0.045	0.179	0.578	0.178	0.601
Caudate nucleus	0.429 (0.059)	0.411 (0.066)	0.436				
Putamen	0.535 (0.056)	0.552 (0.048)	0.302				
Pallidum	0.053 (0.015)	0.063 (0.013)	0.044	− 0.351	0.263	− 0.348	0.294
Amygdala	0.232 (0.025)	0.231 (0.016)	0.931				
Cerebellum	5.196 (0.551)	5.478 (0.518)	0.130				
Third ventricle	0.125 (0.050)	0.097 (0.030)	0.033	− 0.142	0.659	− 0.146	0.669

## Discussion

By analyzing the peripapillary OCT images using a standardized GEE model, we demonstrate that pRNFL thickness is reduced in PSP compared to healthy controls, and correlates with disease stages and some nonmotor symptoms. These results suggest that thinner RNFL can be a potential indicator of PSP progression.

Our findings on pRNFL thinning in PSP patients are consistent with the previous studies. Gulmez Sevim et al. [11] reported that pRNFL thinning was significant in the superior quadrant in 10 eyes of 10 PSP patients compared to 29 PD patients and 33 controls. Another study reported that pRNFL was thinner in 20 eyes of 11 PSP patients compared to 12 PD patients and 12 controls, with no significant difference at the quadrant level [12]. Stemplewitz et al. [13] found pRNFL thinning in the nasal and inferotemporal quadrants in 21 patients with PSP compared to 124 controls, but no correlation was found with clinical severity as assessed by the PSPRS. Compared to the previous studies, the current study data is more robust, because we adopted the standard multivariable GEE model that enables to control the effect of intra-subject inter-eye correlation as well as other covariates potentially influencing the pRNFL measures. This study also first presents the correlation between pRNFL and HY stages in PSP. For diseases with heterogeneous phenotypes, such as PSP, clinical severity is not a direct function of disease duration, which makes HY a more robust indicator of disease severity than simple disease duration.

Increasing evidence suggests that the retina may reflect the brain pathology in central neurodegenerative disorders. In-vivo laser scanning ophthalmoscopic observation of a tau P301S transgenic mouse model, expressing mutant human *MAPT* that induces deposition of 4-repeat tau isoforms in the central nervous system [25], revealed a longitudinal increase in fibrillar tau aggregates in retinal ganglion cells [26]. Postmortem retina of two PSP patients showed hyperphosphorylated tau immunoreactivity in the inner plexiform and ganglion cell layers [26]. Similarly, Lewy-type pathologies have been described in the postmortem tissue of PD retina [27, 28], which well correlated with the severity of brain Lewy body pathologies and the parkinsonian motor symptom severity in the PD patients [28]. In the postmortem retinal tissue of AD patients, extensive axonal immunopositivity for hyperphosphorylated tau was found in the inner plexiform layer, although no fibrillar tau was observed [26, 29]. These previous observations suggest the possible retinal tau pathology in PSP, but there is scarcity of retinal pathological studies in PSP, and the relationship of the retina pathology with brain degeneration in PSP has been unrevealed. In this aspect, our findings are likely to support the assumption that retinal nerve fibers undergo concomitant degeneration in relation to disease progression in PSP, which is worth being confirmed by a large longitudinal study in the future.

Whether the retina is primarily involved in PSP, or the pRNFL thinning reflects a retrograde synaptic degeneration secondary to brain atrophy remains unclear. However, no correlation was observed between the pRNFL thickness and brain volumes in this study, which indicates that retinal nerve fiber degeneration in PSP does not simply reflect brain degeneration in a retrograde manner. Indeed, clinical studies report that visual complaints often occur early in the course of PSP and may even occur prior to diagnosis [2, 3]. In a record-based study in 187 PSP cases, blurred vision or diplopia was recorded to be present in 39%, and photophobia in 20%, whereas visual symptoms were not documented in more than half of the case records [2]. The prevalence increased to 61% for diplopia or blurred vision and 43% for photophobia in 49 patients who underwent standardized clinical evaluation in the same study [2]. A recent retrospective study of 50 PSP and 50 PD patients for prediagnostic symptoms, defined as occurring at least 1 year prior to the diagnosis, showed prediagnostic visual symptoms in 34% of PSP patients, which was significantly higher than 8% in PD patients [3]. Symptoms in the visual domain included diplopia, blurred vision, photophobia, burning eye, and nonspecific visual symptoms, respectively, present in 10%, 8%, 2%, 10%, and 14% in patients with PSP [3]. While oculomotor abnormalities may explain diplopia among these symptoms, the presence of blurred vision, photophobia, and nonspecific symptoms that cannot be explained solely by the supranuclear ophthalmoplegia may be associated with visual afferent dysfunctions, especially involving the non-image-forming visual system [30].

The possible correlation between pRNFL thinning and sleep disturbances in our PSP patients may be worth being noted, although we could not demonstrate the significance after adjusting for multiple comparisons of 28 PSPRS items. In PD, it has been suggested that sleep and circadian disturbances are related to loss of melanopsin retinal ganglion cells that mediate the non-image-forming visual afferent signals [31, 32]. Our exploratory analysis on the correlation between sleep disturbances and pRNFL thinning in PSP suggest a possibility that retinal signaling alterations might play a role in PSP-related circadian problems as they do in PD.

The trend of pRNFL thinning in the inferior segment, as well as the nasal pRNFL thinning correlating to HY stages, suggests a preferential involvement of magnocellular retinal ganglion cell axons in the peripheral retina in PSP. The magnocellular cell (M-cell) axons from the peripheral retina converge through the superior, inferior, and nasal peripapillary quadrants, whereas the maculopapillar bundle fibers enriched in parvocellular cell (P-cell) axons are arranged temporally in the optic nerve head, running horizontally from the nasal side of the macula [33]. In the comparison of macular OCT scans in 22 PSP patients and 151 controls, Stemplewitz and colleagues found that while the superior, temporal, and inferior macular sectors were thinner in the PSP patients, the central nasal macular thickness was not significantly reduced, and the peripheral nasal macular sector was thicker in the PSP patients [13], which may also represent the relative sparing of maculopapillar bundle fibers in PSP. In contrast, PD retinas are presumed to show preferential loss of the parvocellular cells which is represented by temporal peripapillary thinning [33]. In AD, pRNFL is thinner in the superior and inferior quadrants, relatively spared in the temporal quadrant, and hyperphosphorylated tau signals are most apparent in the peripheral retina by a postmortem study [10, 29]. The pRNFL thinning pattern observed in PSP appears to be closer to that in AD. This suggests M-cells that transmit achromatic, motion detection information may be more affected in PSP, which could also contribute to the visual and oculomotor manifestations in PSP.

Some limitations of this study should be discussed. First, our examination was limited to the peripapillary area containing only the retinal nerve fibers. Further analysis of the whole retinal layers, along with the nerve fibers, is required to confirm the retina changes in PSP. Second, the limited number of participants in this study requires careful interpretation of the results. However, to secure statistical reliability, we used age-matched controls and applied a standardized GEE model [14]. Still, no significant difference was found in the pRNFL thickness among the PSP subtypes, or comparison between the healthy controls and the PSP-RS subgroup. We speculate that this may be due to the relatively large number of early staged patients in the PSP-RS group in our study, since 5 out of 15 eyes (33.3%) in the PSP-RS group belonged to patients in HY 4, whereas 3 out of 5 eyes (60%) in the PSP-P group belonged to those in HY 4. The small number of PSP-P patients included in this study further limits the power of subgroup analysis. A large PSP cohort study could elucidate the subtype-specific pRNFL alterations in the PSP population in the future. Third, this cross-sectional analysis result should be confirmed by a longitudinal investigation of ophthalmologic and clinical progression, possibly with comparisons to PD cohorts and with post-mortem investigations, which will validate the usefulness of pRNFL measure in PSP.

The present study is the first investigation of the relationship between retinal nerve fiber degeneration, disease severity with both motor and nonmotor aspects, and brain atrophy in PSP. We demonstrated that pRNFL thickness is reduced in PSP in association with the disease stages and correlated with nonmotor features including sleep disturbances in particular, thereby suggesting the OCT measure as a candidate marker of this neurodegenerative disorder. The lack of correlation between pRNFL thickness and brain volumes and the PSPRS may indicate that the retina is primarily involved in PSP undergoing degeneration in parallel to the brain atrophy. Future investigation of the retinal layer changes in a large longitudinal sample is required to confirm the characteristics of retinal involvement, its relationship with sleep cycle disturbances, and the clinical usefulness of OCT in PSP.

## Supplementary Information

Below is the link to the electronic supplementary material.

Supplementary file1 (DOCX 16 kb)

## Data Availability

The data that support the findings of this study are available on request from the corresponding author.
