# Supplementary material for: Peripapillary retinal nerve fiber layer thinning in patients with progressive supranuclear palsy
Source: J Neurol. 2021 Dec 18;269(6):3216–25. doi: 10.1007/s00415-021-10936-5 (PMC9120117; doi:10.1007/s00415-021-10936-5)
Supplement: Supplementary file 1 — Supplementary file1 (DOCX 16 kb) [file 415_2021_10936_MOESM1_ESM.docx]

**Supplementary Table 1.** Overall results of exploratory correlation analysis between global pRNFL thickness and individual items of PSPRS.

| PSPRS item | *r* | *p* value |
| --- | --- | --- |
| 1. Withdrawal | -0.080 | 0.729 |
| 2. Irritability | -0.359 | 0.110 |
| 3. Dysphagia for solids | -0.124 | 0.593 |
| 4. Using knife and forks, buttoning cloths, washing hands and face | -0.169 | 0.464 |
| 5. Falls | -0.197 | 0.391 |
| 6. Urinary incontinence | -0.472 | 0.031^a^ |
| 7. Sleep difficulty | -0.561 | 0.008^b^ |
| 8. Disorientation | 0.123 | 0.595 |
| 9. Bradyphrenia | 0.076 | 0.744 |
| 10. Emotional incontinence | -0.244 | 0.286 |
| 11. Grasping/imitative/utilizing behaviour | 0.316 | 0.163 |
| 12. Dysarthria | 0.221 | 0.335 |
| 13. Dysphagia | 0.206 | 0.369 |
| 14. Voluntary upward command movement | -0.066 | 0.776 |
| 15. Voluntary downward command movement | 0.006 | 0.979 |
| 16. Voluntary left and right command movement | -0.129 | 0.576 |
| 17. Eyelid dysfunction | -0.026 | 0.912 |
| 18. Limb rigidity | -0.012 | 0.959 |
| 19. Limb dystonia | 0.337 | 0.135 |
| 20. Finger tapping | 0.196 | 0.395 |
| 21. Toe tapping | 0.193 | 0.403 |
| 22. Apraxia of hand movement | 0.038 | 0.871 |
| 23. Tremor in any part | 0.107 | 0.644 |
| 24. Neck rigidity or dystonia | -0.256 | 0.263 |
| 25. Arising from chair | -0.095 | 0.683 |
| 26. Gait | 0.139 | 0.549 |
| 27. Postural instability | -0.128 | 0.581 |
| 28. Sitting down | 0.082 | 0.725 |

^a,b^ With Bonferroni correction for multiple comparisons (x28), these two items did not reach significant level.
